# Distribution and activity of diazotrophs in the Eastern Equatorial Atlantic

**DOI:** 10.1111/j.1462-2920.2008.01796.x

**Published:** 2009-04

**Authors:** Rachel A Foster, Ajit Subramaniam, Jonathan P Zehr

**Affiliations:** 1 Ocean Sciences, University of CaliforniaSanta Cruz, CA 95064, USA; 2 Lamont Doherty Earth ObservatoryPalisades, NY 10964, USA; 3 Presently at the National Science FoundationArlington, VA 22230, USA

## Abstract

The gene abundance and gene expression of six diazotroph populations from the Eastern Equatorial Atlantic in June 2007 were examined using *nifH* gene quantitative polymerase chain reaction (q PCR) methods. Of all the diazotrophs, *Trichodesmium* spp. was the most abundant with the highest number of gene copies in the Gulf of Guinea. *Trichodesmium* also had the highest nitrogenase gene transcript abundance overall with the maximum in samples collected at the equator and in waters influenced by the Congo River plume (> 10^5^ cDNA *nifH* copies l^−1^). Both cyanobacterial unicellular groups (A and B) were detected, where group A was the second most abundant in surface samples, in particular at the stations along the equator. Transcript abundance for group A, however, was at the detection limit and suggests that it was not actively fixing N_2_. *Trichodesmium* and group B *nifH* gene abundances co-varied (*P* < 0.0001). *Richelia* associated with *Hemiaulus hauckii* diatoms were detected in 9 of 10 surface samples and the highest abundances (> 10^4^*nifH* copies l^−1^) were found north-west of the Congo River plume. In contrast, the *Calothrix* symbionts (het-3) of *Chaetoceros* had low abundances at the surface, but were present at 3.7 × 10^4^*nifH* copies l^−1^ at 40 m depth in the equatorial upwelling. This is the first report of the *Calothrix* symbiont in the Atlantic Ocean. This is also the first report of *nifH* gene copy and transcript abundance in an Equatorial upwelling zone. Although the number of gene copies for *Richelia* associated with *Rhizosolenia* were the lowest, the transcript abundance were high (9.4 × 10^1^−1.8 × 10^4^ cDNA *nifH* copies l^−1^) and similar to that of *Trichodesmium*. The distribution of the diazotroph groups, especially the three strains of symbiotic cyanobacteria, was different, and appeared largely controlled by riverine inputs and upwelling.

## Introduction

The Eastern Equatorial Atlantic (EEA) Ocean 10°W−12°E, 6°N−10°S is a region of intense upwelling and where the second largest river in the world, the Congo, enters the ocean. The Congo plume is characterized by warm temperatures, low salinity, low total alkalinity, reduced partial pressure of carbon dioxide (pCO_2_) (Bakker *et al*., 1999), high absorption due to coloured dissolved organic matter (CDOM) and relatively high silicate concentrations compared with the surrounding surface oceanic waters (A. Subramaniam, unpublished). The Gulf of Guinea is also a region of intense precipitation with as much as 30 cm of rain falling per month during the rainy season (Yoo and Carton, 1990). Thus, the EEA is a highly complex hydrographic ecosystem, largely influenced by the Congo River, high precipitation, and intense seasonal coastal and equatorial upwelling in the boreal summer.

Although primary productivity and the nutrient regime of the equatorial Atlantic have been described by several authors in the past (Voituriez and Herbland, 1979; 1981; Herbland *et al*., 1985; Le-Bouteiller, 1986), we have very little information on the actual species composition or functional groups of phytoplankton in these waters. There is detailed information on the species composition in the southern edge of the equatorial Atlantic from a series of transects between 5°S and 15°S from 4°E to the African coast in the spring of 1968 (An, 1971; 1973; Tarkhova, 1973). *Hemiaulus hauckii* was reported to be the dominant member of the phytoplankton community in the southern region of the Gulf (10–15°S), and less common, but abundant were several species of *Rhizosolenia* and *Chaetoceros* diatoms. All three of these diatoms associate with symbiotic heterocystous cyanobacteria, *Richelia intracellularis* and *Calothrix rhizosoleniae.* It should be noted that in these reports, only the hosts were recorded in the observations. There are no observations of the *Chaetoceros–Calothrix* symbiosis in the subtropical and tropical Atlantic Ocean, and some have suggested its geographical limitation to the Pacific and Indian Ocean basins; however, we cannot discount that few have actually looked. Without epifluorescence the inconspicuous character of the symbionts associated with the hosts makes them difficult to see and quantify using microscopy. *Oscillatoria thiebautii* (*Trichodesmium*) was also reported, being more abundant in the coastal eastern (10^3^−10^5^ cells m^−3^) region of the survey than in the western region (10–10^3^ cells m^−3^) (An, 1971; 1973). These free-living and symbiotically associated cyanobacteria populations are known to fix nitrogen and are considered some of the most important diazotrophs in the open ocean (Janson *et al*., 1999).

Recent biological-physical modelling efforts predicted high and persistent *Trichodesmium* abundances and nitrogen fixation in the Gulf of Guinea (Hood *et al*., 2004). In the western tropical North Atlantic (WTNA), where the Amazon River plume enters, a cascade of diazotrophic populations from symbiotic to colonial free-living, to singlet free-living cyanobacteria was described, presumably influenced by the ageing and mixing of the river nutrients (Foster *et al*., 2007). Thus, we were curious if a similar pattern would occur within the region of the Congo River plume and in the EEA. Here we present the abundance and gene expression (*nifH*) for the major open ocean diazotrophs, including *Trichodesmium*, the three symbiotic cyanobacterial strains which associate with diatoms, and two other free-living phylotypes (group A and B) using quantitative polymerase chain reaction approaches (q PCR and q RT-PCR).

## Results

### Surface temperature and chlorophyll as described by remote sensing

**Fig. 1:**
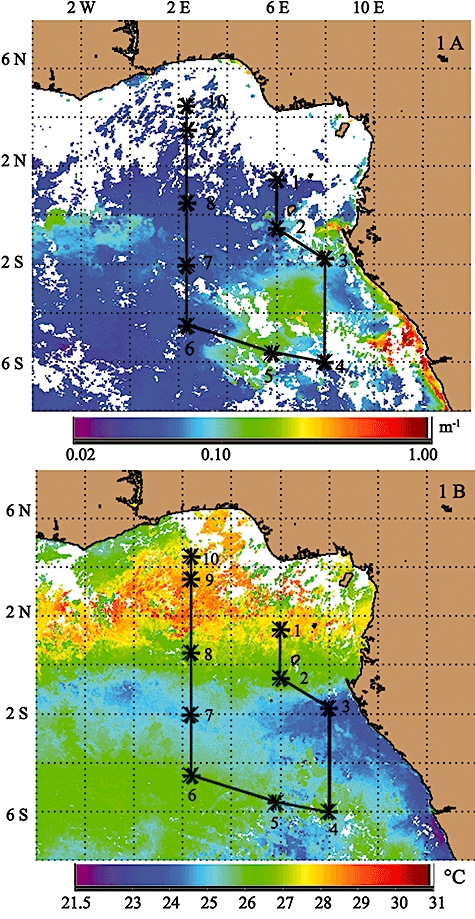
Surface temperature and diffuse attenuation coefficient determined by remote sensing. A. Monthly MODIS composites of K490. B. Night-time sea surface temperature.

Monthly composites of the diffuse attenuation coefficient (K490; Fig. 1A) and night-time sea surface temperature (NSST; Fig. 1B) derived from the MODIS Aqua satellite sensor were used to study the regions influenced by upwelling (cold SST, high K490), the Congo plume (warmer SST, high K490) and precipitation (very warm SST, low K490). The *in situ* temperature and salinity are reported in Table 1. K490 rather than the standard chlorophyll product was chosen for our analysis because absorption due to CDOM was found to be very high in the river plume and upwelling zones causing an overestimation of phytoplankton biomass (A. Subramaniam, unpublished). Stations 1 and 9 appear in the middle of the Gulf of Guinea in very warm waters (> 28°C) with relatively low salinity (between 33.4 and 34.6) representing a region of strongly stratified waters with shallow mixed layer depths, high light, and influenced by high precipitation. Stations 2, 3 and 7 had cooler (around 25°C), more saline (between 35.6 and 36) waters representing the upwelling region. Stations 4 and 5 had relatively intermediate temperatures (around 26.5°C), but low salinity (33.1–33.9) representing the Congo River plume. Stations 6 and 8 also had intermediate temperatures (around 26.5°C) but had higher salinities and seem to be on the outer edges of the equatorial upwelling, representing transitional zones where the upwelled waters mix with typical oligotrophic waters.

**Table 1:** Summary of q PCR assay results from June 2007 samples.

Station	D (m)	T (°C); S (‰)	*Trichodesmium*	*Richelia–Rhizosolenia* (het-1)	*Richelia–Hemiaulus* (het-2)	*Calothrix–Chaetoceros* (het-3)	Group A	Group B
1	1.3	28.0; 33.4	2.1 × 10^4^ (2.9 × 10^3^)	nd (nd)	1.86 × 10^2^ (nd)	nd (nd)	nd (nd)	dnq (nd)
	40.4	22.5; 36.3	nd (nd)	nd (nd)	nd (nd)	5.5 × 10^2^ (3.6 × 10^2^)	nd (nd)	nd (nd)
2	1.2	25.6; 35.6	4.6 × 10^2^ (1.7 × 10^5^)	nd (nd)	8.0 × 10^1^ (8.4 × 10^2^)	nd (nd)	1.1 × 10^4^ (1.3 × 10^3^)	dnq (nd)
	38.2	22.0; 36.2	3.81 × 10^3^ (dnq)	nd (nd)	3.9 × 10^2^ (nd)	6.7 × 10^3^ (nd)	4.2 × 10^2^ (nd)	nd (nd)
3	0	25.1; 36.2	dnq (dnq)	3.8 × 10^2^ (nd)	2.1 × 10^3^ (4.0 × 10^3^)	1.6 × 10^3^ (4.7 × 10^2^)	1.2 × 10^4^ (4.1 × 10^3^)	nd (nd)
4	1.7	26.7; 33.8	8.0 × 10^3^ (1.0 × 10^5^)	dnq (nd)	nd (dnq)	5.6 × 10^2^ (nd)	dnq (nd)	nd (nd)
	29.3	26.4; 33.9	8.3 × 10^1^ (nd)	nd (nd)	2.3 × 10^2^ (nd)	1.0 × 10^2^ (nd)	nd (nd)	nd (nd)
5	0	26.5; 33.1	2.5 × 10^4^ (1.7 × 10^5^)	1.1 × 10^4^ (6.3 × 10^2^)	3.3 × 10^3^ (1.5 × 10^3^)	nd (nd)	7.0 × 10^2^ (dnq)	nd (nd)
6	0	26.5; 34.9	1.4 × 10^4^ (4.1 × 10^3^)	1.3 × 10^3^ (1.8 × 10^4^)	4.3 × 10^3^ (1.8 × 10^4^)	nd (nd)	nd (nd)	nd (b)
	39.3	19.4; 36.0	dnq (nd)	nd (nd)	2.3 × 10^2^ (nd)	4.6 × 10^3^ (nd)	nd (nd)	nd (nd)
7	0	25.3; 35.8	3.7 × 10^2^ (2.5 × 10^3^)	8.7 × 10^1^ (1.4 × 10^3^)	5.0 × 10^1^ (2.8 × 10^3^)	nd (nd)	9.5 × 10^3^ (dnq)	dnq (nd)
	38.8	21.5; 35.8	3.7 × 10^1^ (nd)	4.2 × 10^1^ (4.2 × 10^2^)	3.0 × 10^2^ (nd)	4.2 × 10^3^ (nd)	7.2 × 10^3^ (dnq)	nd (b)
8	0	27.0; 34.7	1.7 × 10^4^ (7.1 × 10^3^)	nd (3.4 × 10^2^)	3.9 × 10^2^ (4.8 × 10^3^)	dnq (nd)	2.7 × 10^4^ (1.1 × 10^4^)	2.0 × 10^2^ (a)
	16.5	26.9; 34.7	2.1 × 10^3^ (1.1 × 10^3^)	nd (8.8 × 10^2^)	5.3 × 10^2^ (3.6 × 10^3^)	dnq (nd)	5.9 × 10^3^ (dnq)	3.8 × 10^1^ (a)
	36.0	19.6; 36.0	1.2 × 10^1^ (nd)	nd (nd)	dnq (nd)	nd (nd)	nd (dnq)	dnq (b)
9	1.2	28.2; 34.6	4.6 × 10^4^ (1.8 × 10^2^)	nd (9.4 × 10^1^)	5.7 × 10^2^ (3.1 × 10^3^)	1.3 × 10^3^ (nd)	nd (dnq)	2.0 × 10^3^ (a)
	19.9	28.2; 34.6	2.1 × 10^3^ (4.2 × 10^3^)	nd (1.2 × 10^2^)	5.0 × 10^3^ (8.1 × 10^3^)	1.4 × 10^3^ (nd)	dnq (nd)	5.2 × 10^3^ (a)
	39.3	25.4; 35.9	6.7 × 10^3^ (2.5 × 10^3^)	3.9 × 10^1^ (4.2 × 10^2^)	9.1 × 10^1^ (6.6 × 10^3^)	nd (nd)	4.5 × 10^3^ (2.0 × 10^3^)	3.3 × 10^2^ (a)
10	1.1	27.8; 34.6	6.1 × 10^4^ (5.7 × 10^3^)	1.7 × 10^3^ (5.6 × 10^2^)	6.6 × 10^1^ (9.6 × 10^3^)	1.4 × 10^3^ (nd)	nd (nd)	3.2 × 10^3^ (a)
	18.3	27.8; 34.6	3.1 × 10^4^ (1.2 × 10^3^)	6.0 × 10^2^ (6.7 × 10^2^)	4.2 × 10^2^ (7.7 × 10^2^)	nd (nd)	dnq (nd)	9.0 × 10^2^ (a)

a Quantitative RT-PCR not processed since samples only collected in the daytime; see *Results* for details.

Mean *nifH* gene copy abundances are shown with transcript abundances for each target in parentheses. nd, not detected; dnq, detected not quantifiable; D, depth; T, temperature; S, salinity.

### Quantitative PCR

The spatial distribution of all six *nifH* phylotypes was examined in surface samples (0–2 m) from 10 stations. In addition, at 3 of these 10 stations, samples were examined for *nifH* gene copy and transcript abundances at mid-depth (16–19 m). Lastly, at 7 of the 10 stations, samples were collected from 29–40 m depths for the same analyses. Since there is considerable evidence that demonstrates that group B temporally segregates its maximal *nifH* gene expression to the night-time (Church *et al*., 2005a,b; T. Shi, unpublished) and given that samples were collected during the daytime, unicellular group B was not analysed for transcript abundance. This was first confirmed in 10 of 20 RNA samples, which were analysed by q RT-PCR for group B transcript abundance and had no detection of transcripts (Table 1).

**Fig. 2:**
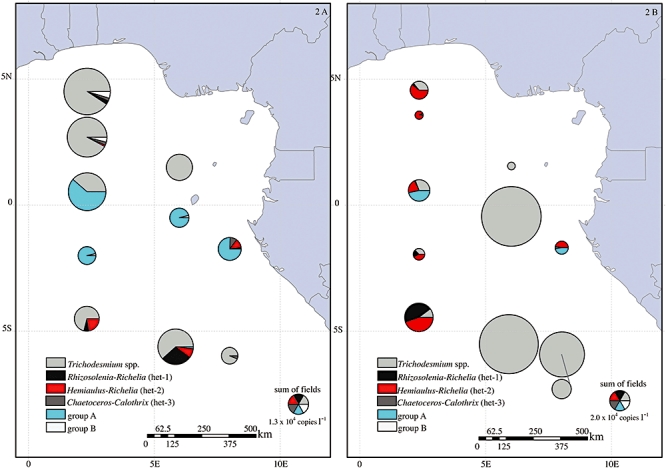
Results from q PCR assays from surface samples collected in the Gulf of Guinea in June 2007. A. The abundance of *nifH* gene copies l^−1^ from q PCR assays. B. Results from the q RT-PCR assays for determining *nifH* gene expression (cDNA *nifH* copies l^−1^). Note that since only daytime samples were retrieved, group B was not included in the transcript abundance analyses.

*Trichodesmium* and group A populations dominated in the surface samples, where *Trichodesmium* had the highest *nifH* copy concentrations (> 10^3^ copies l^−1^) at 7 of the 10 stations (1, 4–6, 9 and 10) and group A was the most abundant at the other 4 stations (2, 3, 7 and 8) (Table 1; Fig. 2A). The high surface densities of *Trichodesmium* were localized to the stations north and south of the equator, whereas stations dominated by group A were along the equatorial upwelling zone (Fig. 2A). Gene expression, however, for group A was not detected or was at the detection limit (> 10 copies l^−1^) and *Trichodesmium* had the highest *nifH* transcript abundance near the Congo River plume and at an equatorial upwelling station (Fig. 2B). There was a significant positive linear correlation between surface *Trichodesmium nifH* abundance and temperature (*r*^2^ = 0.701; *P* < 0.0001).

*Richelia* associated with *Hemiaulus* (het-2) was the most consistently detected of all three symbiotic strains. For example, *Richelia* symbionts of *Hemiaulus* were detected and ranged 50–2.08 × 10^3^ copies l^−1^ in 9 of the 10 surface samples, while the other two strains, *Richelia* associated with *Rhizosolenia* (het-1) and *Calothrix* symbionts of *Chaetoceros* (het-3), were not detected in 4 and 5, respectively, of the 10 surface samples. However, when het-1 and het-3 groups were present, the *nifH* gene copy abundance (> 10^3^ copies l^−1^) was similar to the highest gene copy abundances recorded for *Trichodesmium* and group A. There was no apparent trend in the horizontal distribution of the *nifH* gene abundance for the symbiotic strains and transcript abundance for the symbionts appeared more localized in the surface for stations further off shore.

Unicellular group B was undetected in four surface samples, was detected but not quantifiable in three other surface samples, and was competitively abundant with the other phylotypes in the remaining three surface samples, ranging from 2.04 × 10^2^ to 3.15 × 10^3^ copies l^−1^. When all the *nifH* abundances are pooled, there is a positive and significant correlation between *Trichodesmium* and group B (Pearson correlation, *n* = 10; *P* < 0.0001; *r*^2^ = 0.908) gene copy abundance.

**Fig. 3:**
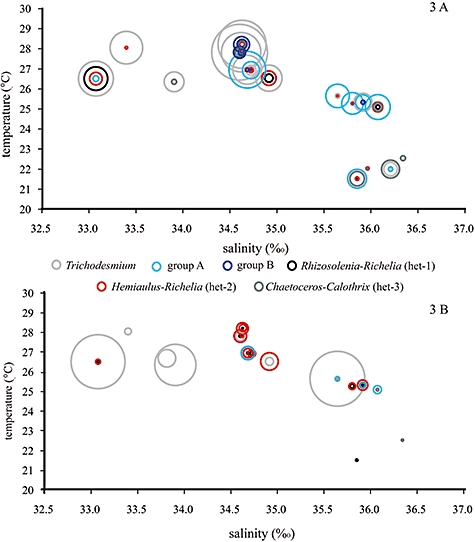
Results from q PCR assays as a function of temperature and salinity. A. The abundance of *nifH* gene copies l^−1^ from q PCR assays. B. Results from the q RT-PCR assays for determining the *nifH* gene expression (cDNA *nifH* copies l^−1^). Note that since only daytime samples were retrieved, group B was not included in the transcript abundance analyses. Scale is relative to abundance of each phylotype where the largest circle represents the highest value in Table 1 surface data.

The gene copy and transcript abundances are plotted as a function of temperature and salinity to identify overlap in abundance for the various phylotypes and to gain better insights into how local hydrography may control distribution and gene expression (Fig. 3A and B). In samples with 35–36‰ salinity and warmer temperatures (26–29°C), the gene copy abundances of *Trichodesmium* and unicellular groups (A and B) overlapped the most; whereas at stations with similar environmental conditions, transcript abundances of unicellular group A coincided with the *Richelia–Hemiaulus* symbioses (Fig. 3A and B). At cooler temperatures (21–26°C) and more saline waters (> 36‰) corresponding to the upwelling region, group A appeared to overlap with the other two symbionts, *Calothrix* and *Richelia* associated with *Chaetoceros* and *Rhizosolenia* respectively (Fig. 3A). These latter relationships, however, did not hold for gene transcription (Fig. 3B).

In the three subsurface samples (16–19 m) collected along 2°E longitude *nifH* gene abundances were highest for *Trichodesmium*, group A and group B (Table 1). In terms of transcript abundance, however, *Richelia* associated with *Hemiaulus* was the highest in two of these three samples (stations 8 and 9), and was nearly equivalent in gene transcript abundances (7.73 × 10^2^ cDNA *nifH* copies l^−1^) with *Trichodesmium* (1.18 × 10^3^ cDNA *nifH* copies l^−1^) at the third station (10). The *Richelia* symbionts of *Rhizosolenia* were within an order of magnitude of the *Hemiaulus–Richelia* symbioses for transcript estimates, and the remaining two phylotypes (group A and *Calothrix*) were either undetected or at the detection limit of the assay (Table 1).

Seven samples were collected from mid-depths (25–40 m) of the upper euphotic zone. The *Calothrix* symbiont of *Chaetoceros* (het-3) was the most abundant in three of these seven samples, where two stations were located in the northern Gulf of Guinea (1 and 2) and the third station was in the western region of the Gulf (station 6) (Table 1). Gene expression for the het-3 group was minimal, as were all the other targets, and was the highest (and detected) only at station 1 (3.6 × 10^2^ cDNA *nifH* copies l^−1^). Similar to its surface abundance, the *nifH* copy abundances for *Trichodesmium* at depth were detected at the highest densities of all targets at stations 2 and 8. *Richelia* associated with *Hemiaulus* (het-2) and group A had the highest *nifH* abundance at the deeper depths at stations 4 and 7 respectively. Group A and *Richelia* associated with *Rhizosolenia* (het-1) were the least detected phylotypes in the subsurface samples (Table 1).

## Discussion

The EEA Ocean has been largely understudied in terms of the structure of the phytoplankton community and microbial activities. Oceanic circulation, on the other hand, is rather well known. The Guinea Current (GC) flows eastward along west Africa and has a strong influence on the surface conditions along the equator, especially during boreal summer when a strong eastward advection of cooler and more saline surface waters enter the Gulf (Richardson and Walsh, 1989). How this hydrography controls the distribution and activity of the phytoplankton community, namely the diazotrophs, was the major focus of this research.

In open ocean environments, *Trichodesmium* spp. are largely considered the most important diazotroph, and at present only nitrogen (N_2_) fixation rates by *Trichodesmium* are included in global nitrogen budgets (Karl *et al*., 2002; Capone *et al*., 2005). More recent studies including this report show that there are other diazotrophic populations that are equally or possibly more abundant. These include contributions by unicellular cyanobacteria, *Crocosphaera watsonii* and the unidentified phylotype group A, and in addition, the heterocystous cyanobacterial symbionts (*R. intracellularis* and *Calothrix compressus*) that associate with diatoms. These other groups can be responsible for a significant portion of the N_2_ fixation in the open ocean (Janson *et al*., 1999; Falcon *et al*., 2004; Montoya *et al*., 2004; Church *et al*., 2005a,b; Zehr *et al*., 2001). Here, we report on all these groups, including group A, which is an uncultivated phylotype, reported from all major ocean basins (Church *et al*., 2005b; Langlois *et al*., 2005; 2008; Foster *et al*., 2007; Man-Aharonovich *et al*., 2007; Zehr *et al*., 2007). *Trichodesmium* was more abundant than all of the other phylotypes, but there were unique patterns of distribution of each phylotype in relation to the hydrography of the Gulf of Guinea.

When interpreting the results from q PCR assays there are a few assumptions to keep in mind. Two important caveats of these approaches are that the gene target exists as a single copy, and second, that there is one genome per cell. At present the number of genomes per cell for each of the targets is unknown. There is some evidence, however, in related heterocystous taxa, e.g. *Anabeana* spp., that multiple genomes per cell exist (Meeks, 2004). In addition, there is one *nifH* copy per genome in vegetative (non-N_2_-fixing) cells and one in the heterocyst. Therefore the estimates for the various heterocystous symbiotic strains (het-1, het-2, het-3) were divided by five as a means to normalize for the multiple copies per symbiont trichome. In the other groups where genomes have been sequenced, in particular *Trichodesmium* and group B (*C. watsonii*), it is known that there is one *nifH* gene per genome. It is also known that DNA and RNA extractions are not 100% efficient and may vary between species. Thus, it is important to note that the gene abundances are approximations and useful for comparing sites but there may be differences in how well *nifH* gene copies estimate cell numbers.

It was interesting and unexpected that the abundance of group A in the surface was the highest along the equatorial upwelling where presumably nutrient concentrations would be higher. Although group A were abundant, their gene transcript abundance at the equator was at the limit of detection, suggesting that this group was not extremely active in N_2_ fixation. The highest concentrations and transcript abundances of *Trichodesmium* spp. were detected in surface samples in the Gulf of Guinea where the model of Hood and colleagues (2004) predicted the highest abundances for the tropical Atlantic. Our abundance estimates for *Trichodesmium* were one to two orders of magnitude higher than those reported in the microscopy cell counts (1–100 cells l^−1^) by An (1971) along the 5°S transect and the pattern of highest transcript abundance for *Trichodesmium* was similar to the cell abundance observations where higher values were recorded near the coast than in the western region. It should be noted that blooms of *Trichodesmium* sp. were also previously observed within the northern regions of the Gulf (Conakry region; 9°31′N, 13°42′W) (Avernia, 1962).

Symbiotic cyanobacteria that reside with diatoms are often overlooked because of their inconspicuous nature. Here we report on the abundance, including transcription, of the *Calothrix* symbiont of *Chaetoceros*. Since the q PCR oligonucleotides target symbiont DNA (RNA) it is possible that the *Calothrix* was present in the free-living state, and therefore the symbioses were not present. However, since few have observed or reported *Calothrix* and the other symbiotic strains (*Richelia*) as free-living (Gómez *et al*., 2005; White *et al*., 2007), we strongly believe that our estimates represent *Richelia* and *Calothrix* in the symbiotic state. *Chaetoceros* diatoms were among the most abundant diatoms reported earlier by An (1971, 1973) in the Gulf. In an earlier study, *Chaetoceros*, *H. hauckii* and *Rhizosolenia* sp. were some of the exclusive diatom populations reported by Avernia (1962) in water samples collected in the Takoradi region (4°55′N, 1°46′W; between Abidjan and Ghana) during the spring and summer of 1960. Semina and colleagues (1976) also reported on a similar species composition in the Gulf of Guinea. They found high abundances of all three diatom hosts and *O. thiebautii* (*Trichodesmium*) in both inshore and offshore waters.

Considering these latter observations, we suggest that the *Chaetoceros–Calothrix* symbiosis was likely present. All other reports of this symbiosis have been limited to the Pacific and Indian Oceans (Norris, 1961; Sournia, 1970; Janson *et al*., 1999; Gómez *et al*., 2005). Although the samples were limited in number, the *Calothrix* symbionts were more abundant and had higher transcription in the deeper samples, suggesting that these associations have a mid-depth optima. Our findings of the *Chaetoceros–Calothrix* symbioses represent the first report of this symbiosis in the Atlantic Ocean.

The *Richelia* (het-1, het-2) associated with *Rhizosolenia* and *Hemiaulus* diatoms were also detected in high abundance and were competitive with the free-living phylotypes in their *nifH* transcription. Since the Gulf of Guinea is quite limited in terms of studies of community structure, in particular symbioses, this too represents one of first reports of these symbioses in the region. At the time of the microscopy observations only the host genera were identified, as without epifluorescence microscopy the symbionts are extremely inconspicuous. Most reports of symbiotic diatoms are in the WTNA (Villareal, 1994; Carpenter *et al*., 1999; Foster *et al*., 2007; Subramaniam *et al*., 2008). Although the sampling was limited, in general the abundance and gene transcription of the symbionts were detected in most samples collected at depth, suggesting that these associations extend to the lower region of the euphotic zone. This was unlike the pattern observed for the group A abundance and gene transcription, where abundance was more consistently detected in the surface samples, and few of the samples (surface and at depth) had transcripts.

The highest abundance of group B was found at stations 8, 9 and 10 in the central Gulf of Guinea with strongly stratified waters, and where some of the highest estimates for *Trichodesmium* spp. were observed. In fact pooling all the data showed that these two populations co-vary. A similar trend was recently reported in samples collected in the WTNA during spring 2003 (Foster *et al*., 2007), and suggests that these populations are capable of co-occurring and potentially occupy the same ecological niches. This latter conclusion is intriguing taking into consideration the differences in cell morphology (unicellular versus colonial), cell diameter and physiological strategies (such as temporal segregation of N_2_ fixation by *C. watsonii*; T. Shi, unpublished), and nutrient scavenging pathways, i.e. phosphorus (Dyhrman and Haley, 2006).

Although our sample collections were limited in number, this is the first report of gene copy abundance and transcript abundance of the *nifH* gene in an equatorial upwelling zone. Thus the traditional paradigm that there is no nitrogen fixation in upwelling regions needs to be re-examined. In addition, we also show that diazotrophs are present and active in gene expression in the Congo River plume potentially supplying new nitrogen to the system, contrary to the conventionally held belief that primary production in river plumes is supported by nitrate supplied by the river that is subsequently recycled. It appears that the distribution and gene expression of these various diazotrophs were different, and were largely controlled by the local hydrography of the region.

Semina and colleagues (1976) hypothesized that the ‘lebensformen’, or lifestyle would dictate the composition of the phytoplankton community. They predicted that species of similar size and morphology would be adapted and coexist under similar hydrographic conditions. For example, the community in the coast contrasts with the composition in the open ocean. Similarly, we found overlapping cell abundances and gene transcription for several groups, including *Trichodesmium* and group B, and group A and diatom hosts with symbionts. As a final comment, the results presented here also demonstrate that even in the background of *Trichodesmium* spp. dominance, there are other populations, e.g. the unicellular groups and diatoms with associated symbionts, which are present and represent an understudied source of new nitrogen and likely an important source of carbon sequestration in the EEA as has been shown for the WTNA (Subramaniam *et al*., 2008).

## Experimental procedures

### Satellite data

The 8-day composite imagery for the period 8–16 June 2007 corresponding to the period of the cruise was obtained from the NASA Ocean colour web (http://oceancolor.gsfc.nasa.gov/). The imagery was processed and the cruise track was superimposed using SeaDAS software (Baith *et al*., 2001).

### DNA and RNA sample collection

Seawater samples were collected along a cruise transect in the EEA (10°W−12°E, 6°N−10°S) aboard the R/V Antea during a research cruise in June 2007. Sampling locations and hydrographic conditions are summarized and shown in Table 1 and Fig. 1. Seawater samples were collected from the near surface and below (16–45 m) using 10 l Niskin bottles arranged on a Conductivity-Temperature-Depth (CTD) rosette. The *in situ* temperature and salinity were recorded from the CTD package. There were a few exceptions. At stations 3, 4 and 5, surface seawater samples were collected with a bucket from the side of the research vessel.

Seawater from the CTD rosette (or surface buckets) was collected into 2 l, bleach-rinsed polycarbonate bottles and immediately 1–2 l was filtered through a 0.2-μm-pore-size Supor filter (Pall Corporation; East Hills, NY) held in a 25-mm-diameter Swinnex filter holder (Millipore; Billerca, MA) using a peristaltic pump. The filters were removed, placed into 1.5 ml bead beater tubes (Biospec Products; Bartlesville, OK), frozen in liquid nitrogen and stored at −80°C until processed in the laboratory for DNA extraction.

In parallel to samples collected for DNA, 2 l of samples were collected for RNA where 1–2 l was filtered as described above for DNA. The filters were placed in 2 ml bead beater tubes (Biospec Products) containing 30 μl of 0.1 mm glass beads (Biospec Products) and 350 μl of RLT Buffer (Qiagen, Valencia, CA) amended with 1% β-mercaptoethanol, froze in liquid nitrogen and stored at −80°C until processed in the laboratory.

### DNA extraction and RNA extraction

The DNA samples collected during the Spring 2007 cruise were extracted using a commercially available kit (Qiagen Plant mini kit). The protocol recommended by the kit was used with modifications of an added 2 min bead beating step, and re-elution with 40 μl of EB buffer (Qiagen). The RNA samples were agitated for 2 min in the bead beater machine, then briefly centrifuged for 2 min and supernatants transferred to clean 1.5 ml microcentrifuge tubes. RNA was extracted following the recommendations of the Qiagen Plant RNA easy kit and protocol. RNA was re-eluted into 35 μl and was subjected to the recommended DNase I step for at least 1 h to avoid DNA contamination.

### Quantitative PCR

In order to determine which populations were actively transcribing *nifH*, total RNA was reverse-transcribed using the Super-Script III cDNA synthesis kit (Invitrogen; Carlsbad, CA) following manufacturer's recommendations. The reaction mixtures contained 2 μl of RNA template, 0.5 μmol l^−1^ of each reverse primers (nifH2 and nifH4) (Zehr and Turner, 2001), 1 mmol l^−1^ dNTP mixture, 1× RT buffer, 5 mmol^−1^ MgCl_2_, 10 mmol l^−1^ dithiothreitol, 1 U RnaseOUT (Invitrogen) and 1 U SuperScript III reverse transcriptase. A second set of reactions was set up as described above, but without SuperScript III reverse transcriptase (RT), which served as the negative controls (No RT). An additional negative control was included which was a water template addition in a reaction with SuperScript III RT and one reaction without enzyme. Reaction conditions were as followed: 55°C for 50 min, 85°C for 5 min, then tubes were placed on ice. One U of RNase H was added to each reaction mixture and tubes were incubated at 30°C for 20 min to eliminate residual RNA. The cDNA was stored at −20°C until used in the q PCR assays.

For the q PCR assays, previously designed TaqMan® (Applied Biosystems; Austin, TX) primers and probes were used to evaluate the *nifH* gene copy abundance for the following target phylotypes: *Trichodesmium*, three symbiotic strains (het-1, het-2 and het-3), and unicellular groups A and B (Church *et al*., 2005a,b; Foster *et al*., 2007).

For all TaqMan® PCR, the 25 μl reactions contained 12.5 μl (13.5 μl) of 2× TaqMan® buffer (Applied Biosystems), 8.0 μl of 5 kDa filtered nuclease-free water (Ambion, Austin, TX), 0.5 μmol l^−1^ each of the forward and reverse primers, 0.25 μmol l^−1^ fluorogenic probe, and 2 μl and 1 μl of template cDNA and DNA respectively. Reactions were performed in quadruplicate, with the fourth replicate used to estimate the reaction efficiency (see below). Two microlitres of 5 kDa filtered nuclease-free water (Ambion) was added for the no template controls (NTCs) and the No RT controls were run the same as the RT samples.

Polymerase chain reaction amplifications were conducted in a GeneAmp 9700 sequence detection system (Applied Biosystems) with the following parameters: 50°C for 2 min, 95°C for 10 min, and 45 cycles of 95°C for 15 s followed by 60°C for 1 min. Gene copy numbers were calculated from the mean C_t_ value of three replicates and the standard curve for the appropriate primer and probe set (see below). In some samples only two of the three replicates produced an amplification signal; these were noted as detectable, but not quantifiable (dnq).

### Standard curves

For each primer and probe set, duplicate standard curves were made from 10-fold dilution series ranging from 10^8^ to 1 gene copies per reaction. The standard curves were made from linearized plasmids containing the *nifH* gene. Regression analyses of the number of cycles (C_t_) of the standard curves were analysed in Excel.

### PCR efficiency

The PCR efficiency for each sample was determined as previously described by Short and colleagues (2004): *X*_*n*_ = *X*_o_ × (1 + *E*_*x*_)^*n*^, where *X*_o_ is the initial number of target molecules and *n* is the number of cycles (C_t_). The *E*_*x*_ is determined by using the calculated *X*_*n*_ with the C_t_ value from the fourth replicate of each sample, which contained 2 μl of the linearized plasmid (10^4^ copies μl^−1^), plus 2 μl of sample DNA. The *E*_*x*_ value was converted to a percentage and samples that amplified with less than 95% efficiency were considered to be inhibited.
